# Comparison of Enzymatic Method Rapid Yeast Plus System with RFLP-PCR for Identification of Isolated Yeast from Vulvovaginal Candidiasis

**Published:** 2011

**Authors:** Moallaei Hossein, Seied Hossein Mirhendi, João Brandão, Reza Mirdashti, Laura Rosado

**Affiliations:** 1 *Department of Medical Microbiology, Faculty of Medicine, Sabzevar University of Medical Sciences, Sabzevar, Iran*; 2 *Departmentof Medical Parasitology and Mycology, School of Public Health and National Institute of Health Researches, Tehran University of Medical Sciences, Tehran, Iran*; 3 *Department of Infectious Diseases, National Iinstitute of Health Dr. Ricardo Jorge, Av. Padre Cruz 1649-016 Lisbon, Portugal*; 4 *Central Labs of Medical Basic Sciences, Faculty of Medicine, Sabzevar University of Medical Sciences, Sabzevar, Iran*

**Keywords:** Candida, Iran, RFLP, Vulvovaginal candidiasis

## Abstract

**Objective(s):**

To compare two identification methods, i.e., restriction fragment length polymorphism (RFLP)-PCR analysis and enzymatic method Rapid TM Yeast Plus System to identify different species causing vulvovaginal candidiasis (VVC).

**Materials and Methods:**

Vaginal discharges of women who had attended the gynecology outpatient clinic of Mobini Hospital in Sabzevar, Iran were collected using cotton swabs and were cultured on Sabouraud dextrose agar. Isolated yeasts were identified by germ-tube testing and Rapid TM Yeast Plus System (Remel USA). For molecular identification, the isolated DNA was amplified with ITS1 and ITS4 universal primers and PCR products digested with the enzyme* Hpa*ІІ followed by agarose gel electrophoresis. Epidemiological and clinical features of women with respect to identified species were also evaluated.

**Results:**

Out of 231 subjects enrolled, 62 VVC cases were detected. The isolated species were identified as follows: *Candida albicans,* 24 (38.7%), *C. glabrata*, 15 (24.2%), *C. kefyr*, 13 (21.0%) *C. krusei,* 9 (14.5%), and *Saccharomyces cerevisi*ae, 1 (1.6%) by RFLP-PCR method; whereas findings by Rapid TM Yeast Plus System were *C. albicans,* 24 (38.7%), *C. glabrata*, 5 (8%), *C. kefyr*, 11 (17.7%) *C. krusei,* 2 (3.2%), *S. cerevisi*ae, 9 (14.5%), and C.* tropicalis, *6 (9.6%) as well as other nonpathogenic yeasts, 4 (6.9%).

**Conclusion:**

Statistical comparison showed that there is no significant difference in identification of C. *albicans* by the two methods; although, in this study, it was not true about other species of yeasts. A correlation between clinical and laboratory findings is important as it enables us to administer an appropriate treatment on time.

## Introduction

Vulvovaginal candidiasis (VVC) is a common problem in women and may affect their physical and emotional health, as well as relationships with their partners (1).

*Candida* species causes 20 to 25% of VVC cases, whereas 40 to 50% of VVC cases are caused by bacteria (2). VVC is characterized by pruritus, soreness, changes in discharges, dyspareunia, vulvar erythema, edema, and fissures (2, 3). The condition is rare before puberty but by the age of 25, nearly 50% of all women will have had at least one clinician-diagnosed episode of VVC (4, 5). The condition is less common in postmenopausal women. Overall, it is estimated that 75% of all women experience an episode of VVC in their lifetime (2, 6, 7).* Candida albicans* is responsible for 78.75% of all cases of VVC in Shiraz, south-west of Iran, (8) and 80 to 92% of all cases of VVC worldwide (2). Vaginal colonization by *non-albicans *species is more common in immunodeficient women. However, an increased frequency of infections by *non-albicans Candida *species, particularly *C. glabrata*, *C. parapsilosis,* and *C. tropicalis* has been reported in healthy women (9). Conventional methods for the identification of *Candida* species are based on assimilation, fermentation reactions, and morphology (10, 11). These techniques are time-consuming and their reliance on phenotypic expression makes them potentially unreliable. Considering many limitations of phenotyping methods for vulvovaginal candidiasis, recent advances in using molecular DNA analysis have facilitated the development of identification systems at species level (10-15). Identification of *Candida *species had been achieved by restriction fragment length polymorphism (RFLP) analysis of the ribosomal DNA (rDNA) repeat of *Candida *species in previous studies (10, 12,16). In the present study, we tried to compare conventional methods, i.e., enzymatic Rapid TM Yeast Plus System with the molecular technique RLFP-PCR.

## Materials and Methods

Having taken written consent, 231 sexually active women participated in this consented study and were examined according to the definition of vulvovaginal candidiasis (severe itching and abnormal discharge). All vaginal specimens were collected in Mobini hospital in Sabzevar, north-east of Iran, between January and December 2007. Vaginal discharge was sampled with two sterile swabs, one swab used for direct examination and 10% KOH and the other one for gram stained smears. The smears were examined for detecting the presence or absence of pseudohyphae and blastoconidia. The other swab was applied to culture the sample on Sabouraud agar medium and then was incubated at 37 ^◦^C for 72 hr.

The pH of vaginal secretions was measured by color pH indicator sticks (MCLB Manufacturing Chemists Inc., Cincinnati, OH) applied directly against the lateral vaginal wall, avoiding contact with cervical mucus. Isolated yeasts were identified based on assimilation and fermentation reactions using Rapid TM Yeast Plus System (Remel USA) morphology and germ-tube testing.

Genomic DNA of each individual fresh colony was extracted and purified using glass bead disruption as described by Mirhendi *et al *(17). Molecular identification was carried out based on the method described previously (18). Briefly, internal transcribed spacer (ITS) regions of ribosomal DNA (rDNA) were amplified using the universal forward (ITS1:5’-TCC GTA GGT GAA CCT GCG G-3’) and reverse (ITS4: 5’-TCC TCC GCT TAT TGA TAT GC-3’) primers.

For the optimum PCR conditions, a reaction volume of 50 µl contained 0.2 mM of each deoxynucleoside triphosphate, (dNTP), 0.5 µM of each primer, 5 µl of 10x PCR buffer and 1.25 U of Taq Polymerase (Fermentas, Lithuania) and 1 µl of extracted DNA as template were used. A negative control containing sterile deionized water instead of the template DNA was included in each PCR run. Reaction mixtures were subjected to an initial denaturation step at 94 ^o^C for 5 min followed by 30 cycles of the following incubations: denaturation at 94 ^o^C for 45 sec, annealing at 56 ^o^C for 1 min and extension at 72 ^o^C for 1 min with a final extension at 72^ o^C for 7 min. A Corrbett research thermal cycler (Australia) was used for the PCR reactions. A 10 µl aliquot of each PCR product was electrophorezed in 1.5% (w/v) agarose gel in TBE (90 mM Tris, 90 mM boric acid, 2 mM EDTA) buffer and stained with ethidium bromide (0.5 µg/ml) and visualized by illumination with UV light. 

For RFLP analysis, 21.5 µl aliquots of PCR products were digested individually with 10 U of restriction enzyme *Hpa*II (Fermentas Lithvania) in a final reaction volume of 25 µl followed by 2.5 hr incubation at 37 ^o^C. RFLP products were analyzed by 2% agarose gel electrophoresis in TBE buffer and stained with ethidium bromide.

Pearson Chi-Square test were used to analyze differences in discrete variables. Findings were considered significant at *P*< 0.05.

## Results

A total number of 231 women were included in this study. The most frequent age interval was between 26 and 35 years old, representing 42% of the total samples. All patients were married, most of them were illiterate (n= 88, 38%) and housewives (n= 222, 96%) and the rate of pregnant women was 33 (14.3%). The mean pH of vaginal samples was 4.79±1.27.

***Direct Examination and Culture***

Sixty two (26.8%) of the women tested positive for *Candida *by culture, of which only 17 (7.4%) were positive by wetmount/Gram stain as a confirmation for vulvo-vaginitis. 

The highest frequencies were found in women between 26 and 35 years, 30 (12.9%) and women between 15 and 25 years old, 21 (9.1%) respectively (Table 2). In addition, from the total yeasts identified 50% and 7.8% were *C. albicans* for symptomatic and asymptomatic vulvovaginal candidiasis respectively.

Seventeen (51.5%) of the pregnant women tested were positive for *Candida* by culture method (Table1).

***Enzymatic Rapid TM Yeast Plus System***

By this method, 24 (10.3%) of isolated *Candida* species were identified as C. *albicans*. Other species, 38 (16.4%), were* C. glabrata*, 5 (8%), *C. kefyr*, 11 (17.7%) * C. krusei,* 2 (3.2%), *S. cerevisi*æ, 9 (14.5%) *C*. *tropicalis, *6 (9.6%), C*. regusa*, *Trichosporon bedgli*, *Blastoschizomyces yesis *and *C*.* elypolytica *each 1 (14.5%), and an unknown yeast 1 (14.5%) (Table 3).

*RFLP-PCR*

**Figure 1:**
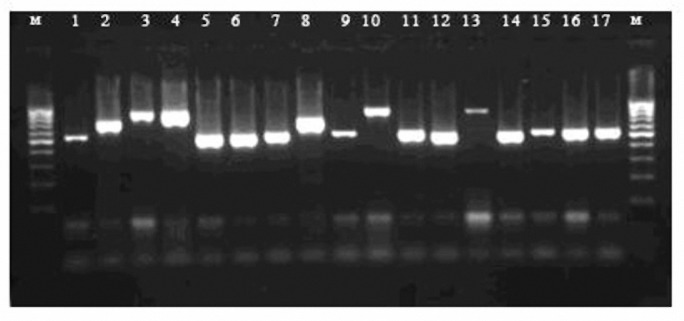
An example of ethidium bromide-stained gel image. Lanes 1-17 amplified ITS region PCR products Lane M: 100 bp ladder molecular size marker. Lanes 1, 59, 11 and 15-17,* C. albicans*; Lanes 2, 7 and 8*C. kefyr*, Lanes 3, 4 and 10 *C. glabrata*; Lanes 6, 12 *C. krusei*; Lanes 13, *Saccharomyces cerevisiae*

**Figure 2:**
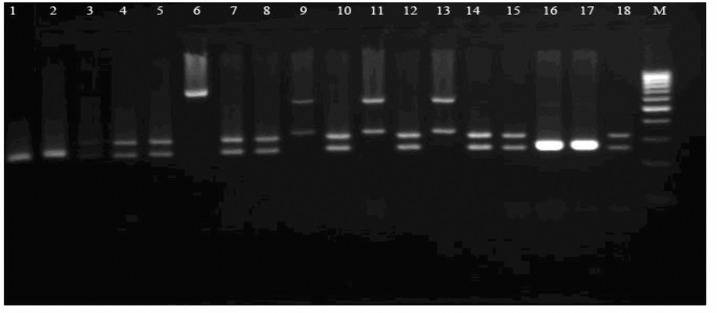
An example of PCR-RFLP patterns for *Candida* isolates Lane M: 100 bp ladder molecular size marker; lanes 1-18. lanes lanes 1, 2, 16 and -17 * C. krusei*; Lanes 9, 11 and 13, *C. glabrata*; Lanes 3 -5, 7, 8, 10,12,14,15 and 18, *C.albicans*; 9; Lane 6 *C. kefyr* .

Fungus-specific universal primer pairs (ITS1 and ITS4) were able to successfully amplify the ITS regions of all tested isolates, providing a single PCR product of approximately 510- 870 bp (Figure 1). PCR products which were digested with *Hpa*II revealed that the bands generated corresponded to the predicated sizes (Figure 2). Digestion of the ITS region of *Candida *species by *Hpa*II generated 2 bands for *C. albicans*, *C. glabrata,* and C*. krusei* (Figure 2). * Hpa*II was not able to digest the ITS regions of *C. kefyr* and *C. parapsilosis*. They were identified using another molecular method (19) (data is not shown). 

**Table 1:** The distribution of different *Candida *species according to the demographic characters by RFLP-PCR method (F= Frequency, Neg. =Negative).

**Demographic** **characterization**	Neg. F %	*positive* F (%)					Total F %
		*C. a lbicans*	Other yeasts				

			*C. glabrata*	*C. krusei*	*C. kefyr*	*Saccharomyces cerevisiæ*	
**Educational standard** Literate I lliterate	72 31.1	16 (7)					88 38.1
		5 (2.2)	3 (1.3)	5 (2.2)	3 (1.3)	0 (0.0)	
	97 41.9	46 (20)					143 61.9
		19 (8.2)	12 (5.2)	4 (1.7)	10 (4.3)	1 (0.4)	
**Occupational** Occupied housekeeper	7 3	2 (0.9)					9 3.9
		0 (0.0)	0 (0.0)	1 (0.4)	1 (0.4)	0 (0.0)	
	162 70.1	60 (26)					222 96.1
		24 (10.4)	15 (6.5)	8 (3.4)	12 (5.2)	1 (0.4)	
**Pregnancy** Pregnant Non-pregnant	17 7.3	16 (7)					33 14.3
		10 (4.3)	4 (1.7)	2 (0.9)	0 (0.0)	0 (0.0)	
	152 65.8	46 (19.9)					198 85.7
		14 (6.1)	11 (4.8)	7 (3.0)	13 (5.6)	1 (0.4)	
Total	169 73.1	62 (26.8)					231 100
		24 (10.4)	15 (6.5)	9 (3.9)	13 (5.6)	1 (0.4)	

**Table 2:** The distribution of different *Candida* species according to age groups. (F=Frequency, eg.= Negative).

**Age groups**	Neg. F %	*positive* F (%)									Total F %
		*C. albicans*		Other yeasts							
				*C. glabrata*		*C. krusei*		*C. kefyr*	*Saccharomyces cerevisiae*		
15-25	46 19.9	21(9.1)									57 24.6
		11 (4.8)	10 (4.3)								
			5 (2.2)		1 (0.4)		4 (1.7)			0 (0.0)	
26-35	67 29	30 (13)									97 42
		8 (3.5)	22 (9.5)								
			10 (4.3)		5 (2.2)		6 (2.6)			1 (0.4)	
36-45	47 20.3	9 (4)									56 24.3
		4 (1.7)	0 (0.0)		2 (0.9)		3 (1.3)			0 (0.0)	
46-55	15 6.4	2 (1)									17 7.4
		1 (0.4)	0 (0.0)		1 (0.4)		0 (0.0)			0 (0.0)	
>55	4 1.7	0 (0.0)									4 1.7
Total	169 73.1	62 (26.8)									231 100
		24 (10.4)	15 (6.5)		9 (3.9)		13 (5.6)			1 (0.4)	

**Table 3:** The distribution of different *Candida species* according to the demographic characters by enzymatic method RapID Yeat Plus System (F- Frequency, Neg.= Negative). 1- *C. glabrata,* 2- *C.tropicalis *3-*C.krusei *4-* C. regusa* 5- *S. cerevisiae* 6- *C. kefyr *7- *T.bedgli *8- *B.capitateis* 9-* C. elypolytica* 10- an unknown yeast

**Demographic characterization**	Neg. F (%)	*Positive* F (%)											Total
		C. *albicans*	Other Yeasts										
			1	2	3	4	5	6	7	8	9	10	
**Educational standard** Literate	72 (31.1)	16 (6.9)											88 (38.1)
		5 (2.1)	1 (0.4)	4 (1.7)	1 (0.4)	0	1 (0.4)	3 (1.2)	1 (0.4)	0	0	0	
	97 (41.9)	46 (19.9)											143 (61.9)
Illiterate		19 (8.2)	4 (1.7)	2 (0.8)	1 (0.4)	1 (0.4)	8 (3.4)	8 (3.4)	0	1 (0.4)	1 (0.4)	1 (0.4)	
**Occupational** Occupied	7 (3)	2 (0.8)											9 (3.9)
		1 (0.4)	0	0	0	0	0	0	0	0	0	1 (0.4)	
	162 (70.1)	60 (9.25)											222 (96.1)
housekeeper		23 (9.9)	5 (2.1)	6 (2.5)	2 (0.8)	1 (0.4)	9 (3.8)	11 (4.7)	1 (0.4)	1 (0.4)	1 (0.4)	0	
**Pregnancy** pregnant	17 (7.3)	16 (6.9)											33 (14.3)
		10 (4.3)	1 (0.4)	1 (0.4)	0	0	3 (1.2)	0	1 (0.4)	0	0	0	
	152 (65.8)	46 (19.9)											198 (85.7)
Non-pregnant		14 (6)	4 (1.7)	5 (2.1)	2 (0.8)	1 (0.4)	6 (2.5)	11 (4.7)	0	1 (0.4)	1 (0.4)	1 (0.4)	
**Total**	169 (73.1)	62 (26.8)											231 (100)
		24 (10.3)	5 (2.1)	6 (2.5)	2 (0.8)	1 (0.4)	9 (3.8)	11 (4.7)	1 (0.4)	1 (0.4)	1 (0.4)	1 (0.4)	

By RFLP-PCR method, *C. albicans,* 24 (10.3%), *C. glabrata,* 15 (6.4%), *C. kefyr,* 13 (5.6%), *C. krusei,* 9 (3.8%), and *S. cerevisiae,* 1 (0.4%) were identified as the etiologic agents of VVC, respectively in the community (Table 1 and Table 2).

## Discussion

Traditionally, identification and characterization of yeast species and strains have been based on morphological traits and especially on their physiological abilities. It is frequently necessary to conduct approximately 5-10 tests to identify reliability at the species level for most of the yeasts (20-22). In contrast, molecular biology techniques provide alternative methods and are becoming routine tools in the identification of yeasts. RFLP-PCR is based on the digestion of amplified DNA. Every organism possesses unique nucleotide sequences that distinguish it from every other organism on the basis of the number and size of the fragments. DNA is extracted from isolates and cleaved into fragments by restriction endonucleases and the fragments are separated by gel electrophoresis (12,15). RFLP requires no long time and work. The method has been used for genotyping a variety of pathogens within the last few years (15).

In this study, the frequencies of isolated yeasts from VVC were studied by a profile of PCR-RFLP previously described by Mirhendi and coworkers (19). 

In the present study, the median age of women, with positive culture was 29.5±6.5 and those with negative culture was 33.8±9.8 and independent samples t-test revealed that this differences is significant and the Pearson’s Chi-square test showed a significant statistical correlation between positive vaginal* Candida* culture, pregnancy and pH of vagina (*P*< 0.005). The overall prevalence of vulvovaginal candidiasis in a community setting was found to be 26.8%. This was higher than Hamadan, northwest of Iran and elsewhere (23-25) and lower than Shiraz and south of Iran (8). Some previous reports rated prevalence between 30 and 50 percent (26). However, the overall percentage of non-albicans vaginitis was 16.4%. Although this data was closed to studies of Spinillo (27), who found 17% for the rate of non-albicans* Candida *species*, *it showed higher rates than those in studies of Vabrik and Pauitch. It was also lower than those in studies of Pakshir (8), Mohanty *et al* (24), Nyirjesy *et al* (30), where these rates were 21.25, 4.8 and 32%, respectively (8).

Comparison between the molecular and enzymatic methods demonstrated that both of them have a similar sensitivity to the *albicans* species of *Candida*; but they are different in others. The molecular method as a gold standard showed that there might be false positive results in identification of non-albicans species such as *C. regusa*, *T. bedgli*, *B. capitateis, C*.* elypolytica, C. tropicalis, *and* C. cerviciea, *and false negative issues in differentiating of species like *C*. *glabrata, C. krusi, *and *C. kefyr.* These suggest that where identification of yeast species, such as antifungal drug administration, is important the molecular method is preferred (Table 2 and Table 3). 

A postulated risk factor for* C. glabrata *vaginitis is a higher alkaline pH. It also occurred in concomitant bacterial vaginitis which was in agreement with our findings (31).

Our results showed that the detection of *Candida* in vaginal swabs is corolated with the age of patients. Some previous reports were in agreement with our results (2, 4-6). It was shown that women under 35 years old (Table 2, Group I and II) have the highest rate of detectable *Candida* compared to the other groups. This rate was undetectable over the age of 55 years (Table 2, Group V). In addition, our data showed that *Candida *species had interesting trends to the age of patients as the overall detection of *Candida* decreased with age and changed in the distribution of individual species. The detection rate of *non-albicans Candida* increased 2.75 folds in the age group of 26 to 35 years (Table 2, Group II). This rate decreased 1.25 folds in the age group of 46 to 55 compared to the positive cases for* C. albicans* in the age group of 55 to 65 years (Table 2, Group III). These results were close to those found by Trama *et al* (32).

Moreover, the rate of *C. glabrata* increased 2folds in the age group of 26 to 35 years (Table 2 Group II). It could be postulated that these changes in distribution were related to selection of resistant species by widespread use of azoles-based anti-fungal drugs (33), hormonal changes due to menopause and estrogen treatments (6,7), the effects of aging on protective immune responses (34,35), and prolonged stays in hospital (33). These factors might change the vaginal environment and the characteristics of vaginal floral to favor the growth and virulence of non-albicans species, or be due to increase exposure of subject to non-albicans species.

We observed that the incidence of VVC in pregnant women was 3.5 fold higher than that of non-pregnant women. It continued to increase in the third trimester of pregnancy which was in agreement with other study (36).

Pregnancy has been known to be associated with depressed aspects of cell-mediated immunity that permit fatal retention. Moreover, the hormonal changed milieu of the vagina during pregnancy enhances *Candida* colonization and serves as a risk factor for symptomatic expression (37).

## Conclusion

According to our findings, RFLP-PCR can be used as a gold standard method for identification of isolated yeast from vulvovaginal candidiasis. Otherwise, the enzymatic method RapID Yeast Plus System is not capable to identify different yeasts. 
